# Dynamics of volcanic ash remobilisation by wind through the Patagonian steppe after the eruption of Cordón Caulle, 2011

**DOI:** 10.1038/srep45529

**Published:** 2017-03-28

**Authors:** Juan E. Panebianco, Mariano J. Mendez, Daniel E. Buschiazzo, Donaldo Bran, Juan J. Gaitán

**Affiliations:** 1 INCITAP and Facultad de Agronomía-UNLPam, Santa Rosa (6300), Argentina; 2 INTA, Argentina

## Abstract

Wind erosion of freshly-deposited volcanic ash causes persistent storms, strongly affecting ecosystems and human activity. Wind erosion of the volcanic ash was measured up to 17 months after the ash deposition, at 7 sites located within the ash-deposition area. The mass flux was measured up to 1.5 m above ground level. Mass transport rates were over 125 times the soil wind-erosion rates observed before the ash deposition, reaching up to 6.3 kg m^−1^ day^−1^. Total mass transport of ash during the 17 months ranged between 113.6 and 969.9 kg m^−1^ depending on topographic location and wind exposure. The vertical distribution of the mass flux at sites with higher vegetation cover was generally inverted as compared to sites with lower vegetation cover. This situation lasted 7 months and then a shift towards a more uniform vertical distribution was observed, in coincidence with the beginning of the decline of the mass transport rates. Decay rates differed between sites. Despite changes over time, an inverse linear correlation between the mass transports and the mass-flux gradients was found. Both the mass-flux gradients and the average mass-transport rates were not linked with shear-stress partition parameters, but with the ratio: ash-fall thickness to total vegetation cover.

Volcanic eruptions can cover landscapes with volcanic ash, creating extensive areas with unstable surfaces^1^. Low density of dry volcanic ash can lower the threshold wind speed required to move the surface material, increasing the sediment transport^2^,^3^. The movement of volcanic material across the surface and the entrainment of the finer fraction into the atmosphere, causes impacts on the health of people, wildlife and livestock; and also damages property^3^,^4^,^5^,^6^,^7^,^8^. At larger scales, volcanic ash present in the atmosphere can affect solar radiation, and the nutrients contained in the ash can fertilize terrestrial and aquatic ecosystems far away from the source, contributing to carbon sequestration in biomass^6^,^9^. Recent research shows that the presence of volcanic ash can also affect important soil chemical properties^10^. However, knowledge of field conditions and wind erosion rates of fresh volcanic deposits is very limited^11^.

On June 4, 2011 the Cordón Caulle, located at 40°32′ south latitude and 72°7′ west longitude in the Republic of Chile, began a new eruption. The explosive eruption dispersed about 1000 million metric tons of ash on 7.5 million hectares of northern Patagonia^12^. The thickness of tephra ranged from 30 cm in the Andean area to less than 1 mm in the Atlantic coast zone. Detailed characteristics of the eruption and dispersion process are provided by Pistolesi *et al*.^13^, and Bonadonna *et al*.^14^. Eruptions of volcanoes located along the Andes and the subsequent impacts of the transport of pyroclastic material by winds from the west over Patagonia have received attention after the eruptions of Hudson volcano in 1991^2^ and Chaitèn volcano in 2008^5^,^6^,^7^,^8^,^15^, but magnitude and characteristics of surface transport rates of the fresh ash over the landscape are still unknown.

With exception of the study that quantified one extreme wind erosion event in Iceland^16^, to our knowledge, studies of the mass transport of fresh volcanic ash at the surface level are non-existent. There is a need to estimate the importance of the resuspension of volcanic ash for posteruptive environmental conditions^17^. Moreover, at present the remobilisation and resuspension of ash can only be analyzed within the soil wind erosion – dust emission framework^18^, and there is a need to assess the dynamics of erosion and re deposition of ash due to discrepancy between the simulation of the remobilisation of ash and field observations^19^. The objective of this study was to describe the aeolian transport of freshly-deposited volcanic ash up to 1.5 m above ground level, at different sites across a Patagonian-steppe landscape during the first 17 months after the ash fall.

## Results and Discussions

### The vertical distribution of the horizontal mass flux

**Figure 1:**
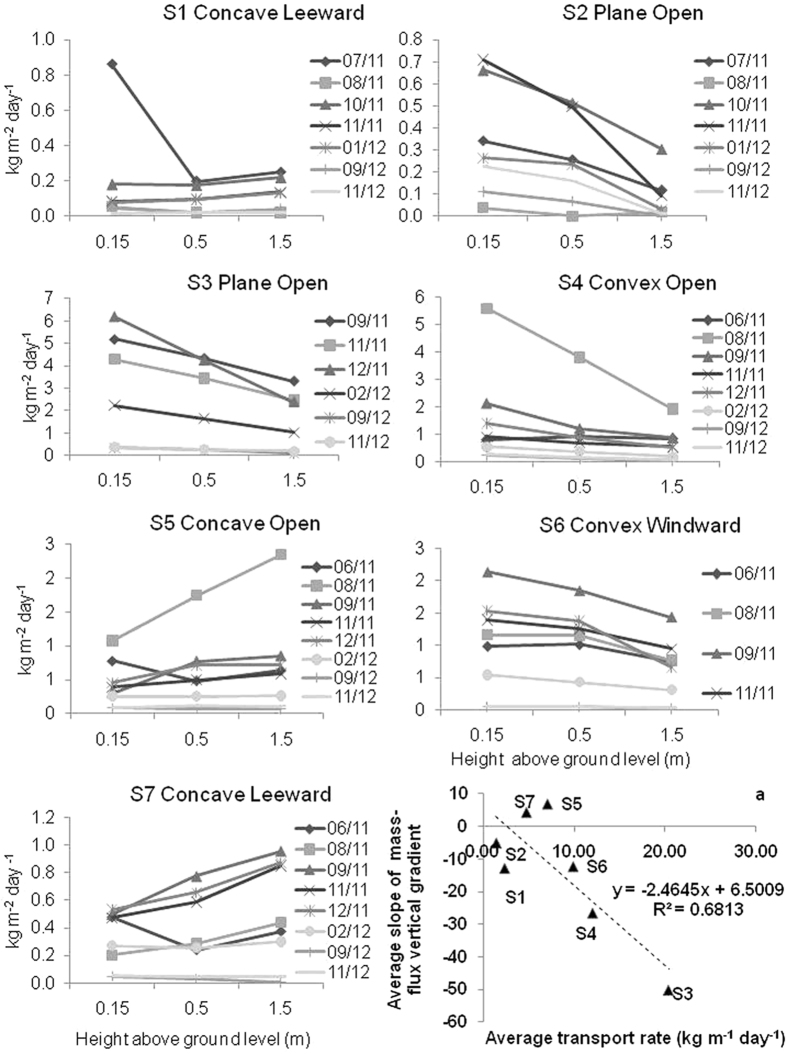
Mass flux gradient at sites 1 to 7 (S1 to S7). Figure 3a shows the linear correlation between the linear slope of the mass flux gradient and the average mass transport rates at the seven sites (p < 0.02). Please note that if every mass flux or mass transport value is considered instead of the averages per site, then the statistical assumption of independency is violated because the events are successive in time.

Figure 1 shows the linear interpolations between the mass fluxes measured at different heights (the mass flux gradient) at sites 1 (S1) to 7 (S7). S1 showed a more homogeneous vertical distribution, except for the first measurement period. Located in a plane open area, S2 showed a more typical vertical mass-flux distribution: a negative slope. By typical we mean that most of the transported mass was concentrated near the surface, as it is usually observed for sand and soil particles on unvegetated wind-eroding fields. At S3, despite the intermediate values of soil cover and flow regime (Table 1) mass fluxes were the highest. We believe this happened because this site was placed in the bottom of a big valley, parallel to the predominant wind direction. This valley acted as a funnel for the aeolian redistribution of the ash at the landscape level, and hence it worked as a preferential path for the transport of ash. At this site, negative slopes were observed throughout the entire measurement period. Westerly sites, S4 and S6, generally showed negative slopes, but S5 and S7 generally showed positive slopes.

Many researchers have found that wind eroded mass flux decays exponentially with height^20^, even for volcanic material^16^. However, several authors have demonstrated that vertical profiles of both the mass flux and the wind speed can be complicated^21^,^22^,^23^,^24^, especially in the presence of vegetation^25^. Herein, the typical negative slope for aeolian mass transport was found at low cover, convex sites (S4 and S6). By typical we mean that most of the transported mass was concentrated near the surface, as it is usually observed for sand and soil particles on unvegetated, wind-eroding fields^20^. But at the concave, and more vegetated, sites (S5 and S7), the mass flux profile was generally inverted respect to the convex sites. The higher coverage and connectivity of the vegetation found at the concave areas (Table 1), caused interference of the horizontal mass flux and also reduced the wind speed below the canopy height^25^, producing different vertical profiles than those observed at sites with less vegetation cover.

During the first sampling period, the profile at the more vegetated place, S1, also showed a decrease of the mass flux up to 0.5 m height, similar to what was observed at S2 (Fig. 1). This exception can be explained by the very large amount of fresh material moving below the canopy height at the beginning of the ash transport process. The differences in the positive slopes between convex sites S4 and S6, can be attributed to the leeward location, and therefore relatively less wind speed at the latter. Despite these particular cases, the differences observed between sites with high vegetation cover (concave, accumulation) and low vegetation cover (convex, deflation) were consistent.

Approaching the end of the sampling period, a clear trend towards a more homogeneous distribution of the mass flux across the height was consistently observed at all the sampling sites. This trend can only be explained by the depletion of the erodible material during the two-year measurement period. Of course other variables could have affected the process in different ways at each site, such as the climate gradient, the heterogeneous redistribution process, the immobilization of the ash in the soil, the water erosion, and the resuspension of the ash. However, all of these variables contributed to the depletion or stabilisation of the ash, therefore decreasing the supply of erodible material at every site. By comparison between the ash depth values measured on november 2011 and the initial values (Table 1), it can be seen that most of the ash was already removed from the inter patch spaces by that date.

The higher concentration of the ash flux closer to the ground, mainly at the convex and plane sites, shows the high stress that people^26^ plants and animals had suffered due to the impacts of the moving particles. Although stress was measured for vegetation^27^, insects^28^, mammals^29^ and reptiles^30^, the mass flux is never mentioned as a cause of damage or stress in the literature. According to our results, sites with higher vegetation cover, especially leeward areas, could provide relatively better sheltering conditions for plants and animals. However, herbivorous insects can be negatively affected for longer periods in the areas where the volcanic ash availability lasts longer^28^, also affecting the food chain in these areas^30^. Animals that are less adapted for locomotion over the ash^30^ and animals feeding on vegetated areas^29^ could also suffer the negative impacts of the presence of ash for longer time at these areas.

### The horizontal mass transport

**Figure 2:**
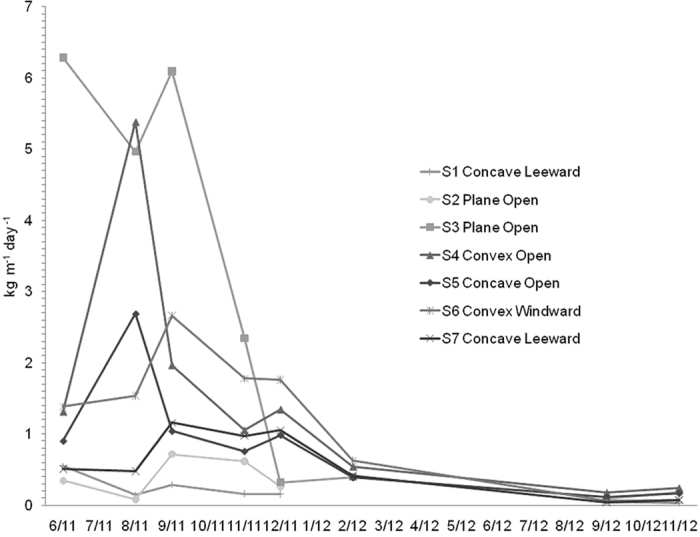
Timeline of the mass transport rates at different sites within the landscape. After six to eight months after the ash deposition, a clear trend towards stabilization of the mass transport rates over the time was observed at every site.

Horizontal mass transport rates per site and date are shown in Fig. 2. Values of the horizontal mass transport rates measured prior to the ash fall were 0.002 and 0.06 kg m day^−1^ for S1 and S2 respectively. After the ash fall, horizontal mass transport values increased up 0.55 kg m day^−1^ S1 and up to 0.72 kg m day^−1^ at S2. Despite the low wind speeds during this period, the greater amount of ash fallen at the site with higher vegetation cover (S1) produced larger mass transport values during the first winter period. The combination of high material availability in the presence of vegetation can produce higher horizontal mass transport rates than the bare surfaces, because of the turbulence produced by vegetation^25^. Despite this, another factor as local rain can have affected this value. However, from the beginning of the windy season in spring, the mass fluxes were higher at the site with degraded conditions (S1), due to the lower vegetation cover and connectivity (Table 1) at this site. The mass transport rate observed 17 months after the ash deposition was 0.03 and 0.19 kg m day^−1^ at S1 and S2 respectively. Total mass transport was 113.6 kg m^−1^ at S1 and 156.8 kg m^−1^ at S2.

The highest mass transport values were observed at S3, reaching 6.3 kg m day^−1^. Unfortunately, mass transport values prior to the ash fall were not measured at this site. Despite the intermediate vegetation cover and flow regime values (Table 1), mass transport rates of volcanic ash were consistently higher at this point of the studied transect. We consider that this happened because S3 was located in a wide, long valley. Much of the ash from the deflation (convex) areas of the landscape was redistributed to lower, accumulation (concave) areas such as valleys. Moreover, this valley was conveniently oriented parallel to the prevailing wind, producing a preferential path for aeolian transport. The mass transport rate observed 17 months after the ash deposition at S3 was 0.4 kg m day^−1^. Total mass transport at S3 was 969.9 kg m^−1^.

The horizontal mass transport values measured prior to the ash fall were 0.09 kg m day^−1^ at the site located in the convex area (S4) and 0.05 kg m day^−1^ at the site in the concave area (S5). After the ash fall, horizontal mass transport values increased up to 5.38 kg m day^−1^ at S4 and to 2.69 kg m day^−1^ at S5. However, the general difference between the mass transport rates at the concave (S1, S5 and S7) and convex (S4 and S6) or plane (S2) areas was consistent. The mass transport rate observed 17 months after the ash deposition was 0.24 and 0.17 kg m day^−1^ at S4 and S5 respectively. Total mass transport was 552.1 kg m^−1^ at S4 and 329.2 kg m^−1^ at S5.

At S6 and S7 the horizontal mass transport values prior to the ash fall were 0.021 kg m day^−1^ at the site placed at the convex area (S6) and 0.019 kg m day^−1^ at the site at the concave area (S7). After the ash fall, horizontal mass transport values increased up to 2.66 kg m day^−1^ at S6 ant to 1.16 kg m day^−1^ at S7. The mass transport rate observed 17 months after the ash deposition was 0.07 kg m day^−1^ at both S6 and S7. Again, the general difference between the mass transport rates at the concave (more vegetation cover) and convex (less vegetation cover) areas was consistent. Total mass transport was 463.7 kg m^−1^ at S6 and 222.2 kg m^−1^ at S7. Despite the differences between sites, a clear trend towards stabilisation of the mass transport rates over the time was observed at every site after 7 months (Fig. 2). Similar stabilisation period was reported after the Hudson eruption in 1991 on a similar landscape^2^.

Although the mass transport values are presented as precise values, the uncertainty in the quantification of the mass transport of wind-eroded sediments is a controversial subject^31^. Considering the BSNE efficiency^32^,^33^ range and the limitations of the methodology^34^ discussed in the methods section, the values presented here could be underestimated by up to around 50%, especially at the plane and the convex areas. However, the main reason for the underestimation in that case was due to the fact that the distribution of the mass flux across height was exponential, which is not the case in most of the datasets observed herein, especially during the periods of greater mass fluxes (Fig. 1). This suggests that underestimates, if any, were not so large. However, the particular characteristics of the vertical distributions discussed in the previous section, as well as their changes over time, makes any attempt of correction also uncertain. Hence, in order to maintain the measurements without additional distortions, we present the results of the original calculations. In any case, the values are still very useful for the relative comparisons between sites and dates, since the methodology of sampling and calculation is robust^34^ and it was constant throughout the study.

### The link between the vertical distribution of the mass flux and the horizontal mass transport

The mass fluxes and the mass transport are closely related because the second one is derived from the integration of the first over the height (Please refer to section 3.4).

It is generally accepted that the gradient of the eroding mass flux above the surface exhibits a negative slope (decreases with height). However, we observed that the vertical distribution of the mass flux differed from one sampling site to another, even changing along the sampling time. From the comparison of the dates and values shown in Figs 1 and 2, it can be deduced that as the horizontal mass transport rates increased (up to September–November 2011), the transported ash was more concentrated near to the ground (negative slope) in convex and plane sites, while the opposite was generally observed at concave sites. Conversely, as the horizontal mass transport rates decreased, the values of the slopes of the vertical mass-flux profiles showed a tendency to increase at the convex and plane sites (opposite to concave sites) (from September- November 2011 to November 2012).

These results have one main implication for the methodology of aeolian mass transport calculation over natural areas: the usual procedure of fitting a fixed function to vertical mass-flux gradients and then integrating over the height is difficult when the profile shapes and slopes are variable. Hence, making assumptions like considering preset vertical distribution coefficients, as it is sometimes done for reducing the sampling points above the ground^11^, or even preset functions, can be misleading.

Despite the changes over time of the mass flux distribution above the ground and the mass transport rates, the average for the entire sampling period produced an inverse linear correlation between the horizontal mass transport rate and the vertical distribution of the mass flux (Fig. 1a). This correlation confirms that, on average, the mass transport rates at the convex and plane open sites where higher than those at the concave sites, especially at the eastern part of the studied area. The average trends and values, as well as the changes over time observed at the different sites can be important for understanding and modelling the remobilisation and resuspension of ash. Until now, these values were generally neglected or roughly estimated, due to the lack of information or to the inability of the models to adequately simulate the time-dependant processes, as well as the effect of other variables that are discussed in the following section.

### Main factors affecting the remobilisation of fresh volcanic ash by wind

At present, the only option for estimating the resuspension of volcanic ash are the models developed for the emission of mineral dust^18^. Aeolian sediment transport models in the presence of vegetation are based on the interaction between the wind speed, the threshold wind speed and the roughness elements^35^,^36^. The roughness elements are generally represented by shear-stress partition parameters (Table 1). The parameters presented in Table 1 were tested for correlation with the average mass transport values, but no significant correlation was found even for the total vegetation cover. We believe this happened because although roughness parameters in these environments do not change through time, other parameters can change significantly.

Although herein the wind was not measured precisely at every site, the general trends observed at every site suggest that additional factors can also be critical for explaining the aeolian mass transport, particularly at the landscape scale. It was previously found that even the shear velocity can be a bad predictor of the mass transport because other key factors can be dynamic^37^, like the changes in the availability of erodible material (supply limitation). Other factors can change during the sampling period too, such as the distance traveled by the wind along the prevailing direction from a non-erodible area up to the sampling point, known as the fetch effect^38^. This can happen because of the heterogeneous redistribution of ash over the landscape (Sections 1.1 and 1.2), producing the accumulation, at certain areas, of material that can be re transported later. Moreover, the amount of ash fallen at different sites (ash depth) was not identical (Fig. 4), hence the availability of erodible material was quite different at certain sampling points from the beginning of the observation period.

### Supply limitation

It is known that wind erosion rates are strongly affected by the availability of erodible material^39^,^40^. The highest horizontal mass transport values were observed during the first windy season. This initial “blow off” lasted seven months. From then on, as the mass transport rates decreased because of the depletion of erodible material, the values of the slopes began to increase towards zero, indicating a more uniform vertical distribution of the mass fluxes. Wilson *et al*.^2^ also reported that the impacts of the remobilization of ash lasted for more than six months after the 1991 Hudson eruption in Southern Patagonia.

Supply limitation is a documented phenomenon, especially under wind tunnel conditions. However research on this subject is scarce^20^. The parameter σ is used for describing supply limited saltation, it indicates the exhaustion rate of the particles that are available to be transported by the wind. In practice, σ is time dependent and it typically decays exponentially^20^. However, fluctuations in the transport rates were observed over the time (Fig. 4), and the decay trends resulted more linear than exponential (regressions not shown in this work). Values of the parameter σ (monthly average) are shown in Table 1. Considering 17 months, the average monthly decay rates of the mass transports were lower at convex and plane sites. However, considering a general average the monthly decay rate was similar: 9.8% and 9.5% for concave and convex or plane - open sites, respectively.

### Topography

**Figure 3:**
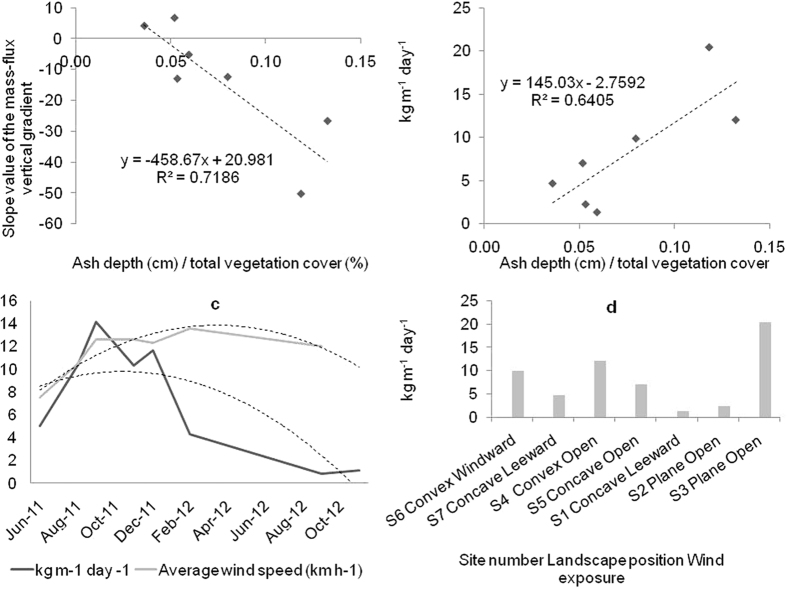
Correlation between ash depth to total vegetation cover ratio and the average slope of mass-flux vertical gradient (p < 0.01) per site (**a**); the average mass transport for the entire sampling period (p < 0.05) per site (**b**). Timeline of average mass transport per period and wind speed (**c**), dotted lines indicate tendencies. Average mass transport during the entire sampling period, according to site location and wind exposure.

The average transport rates of volcanic ash were higher in plane, convex, and windward sites (Fig. 3d). Summits and plane positions had lower rates of dust deposition in Mongolia grasslands^41^. These authors also stated differences in wind speeds between plane, leeward and windward locations. Besides differences in the wind speeds, the relief produces a differential distribution of natural resources such as light, water and nutrients. In convex areas, vegetation cover and connectivity were lower (Table 1), so the ash was rapidly transported by the wind. In concave areas the greater connectivity of the vegetation helped to maintain a reserve of ash on the ground due to the decrease of the wind shear. According to visual observations, part of the ash was trapped in the leeward zone behind the patches of vegetation, and also within the gravel, especially at concave areas. This explains why the mass transport rates at convex sites generally decreased faster than at concave sites (Fig. 2). This result suggests a relocation of the volcanic ash, from convex areas to concave areas of the landscape. However, the deposition of erodible material in large, concave areas oriented parallel to the predominant wind direction can produce larger mass transport rates at some points of the landscape, as we believe it was observed at site 3. The average difference in the transport rates between the plane or convex sites and the concave sites, ranged between 48% and 68%. This implies a potential average retention (or stabilisation) of ash within the studied landscape of 42%.

### Ash depth to vegetation cover ratio

The slope of the mass flux gradient and the average mass transport rate were not only affected by the time elapsed since the ash deposition, but also by site-specific factors, particularly the ash depth at the beginning of the transport process and the vegetation cover (Fig. 3a and b respectively). There are two explanations for differences in mass flux profiles over vegetated areas: the vertical distribution of shear stress velocity, and the selective filtering of particles with relatively small ejection angles^25^. In practice, the particles moving within the canopy height tend to move more slowly, producing a smaller mass flux. The particles that are ejected over the canopy layer move faster, producing a greater mass flux. If material availability is higher, then more particles are potentially available to be ejected and transported. On the other hand, if vegetation cover is dense, the particles are protected from the wind shear and if they move, they do it slowly. No significant correlations were obtained when considering the ash depth and the vegetation cover separately.

## Methods

### Geographical layout and ash deposition

**Figure 4:**
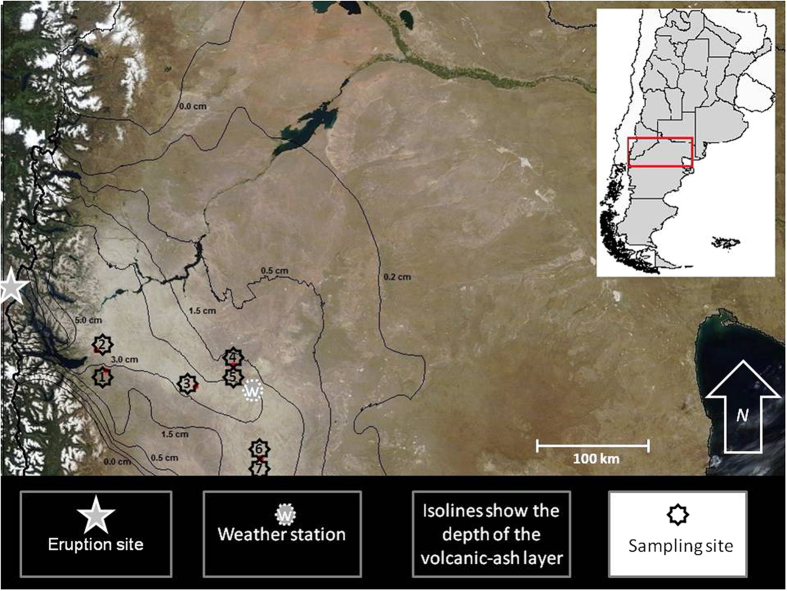
Location of the sampling sites and ash deposition map. The image was taken by MODIS after the ash deposition: Vermote E. (2015). MODIS 8-day Composite MOD09Q1 MODIS/Terra Surface Reflectance L3 Global 250 m SIN Grid V006. NASA EOSDIS Land Processes DAAC. MOD09Q1.h12v13.006 and MOD09Q1.h12v12.006. Day 289, 2011. Isolines were generated from field observations with ArcGis 9.2 geostatistical tool^12^ (http://www.esri.com/software/arcgis).

The study region is placed at the Western Andean region of Northern Patagonia, Argentina. Measurement sites were placed along a 150 km transect, in the context of a rangeland wind-erosion monitoring program. The sampling sites were chosen according to contrasting differences in topography and vegetation condition. After the eruption, volcanic ash covered the sites, as shown in Fig. 4.

**Figure 5:**
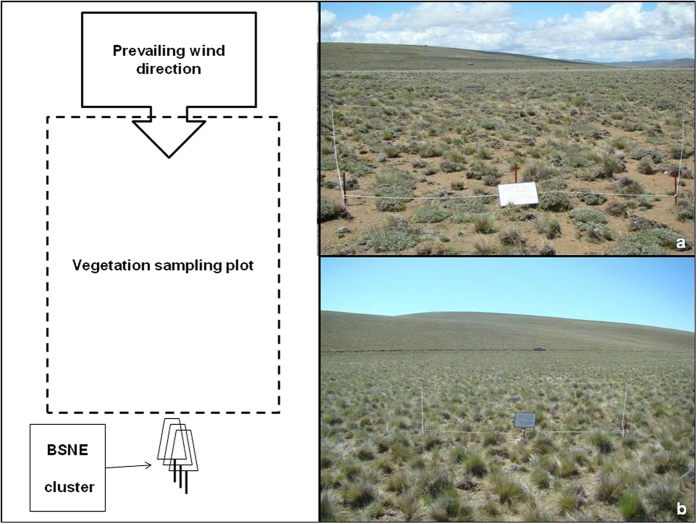
Picture taken at site 2, showing part of a sampling plot (**a**). Scheme of the topographic position and wind exposure of the sampling sites (**b**). A BSNE cluster at site 3, several months after the ash fall (**c**).

The vegetation at the study area is a low, grassy, shrub-steppe. The average annual wind speed for this region ranges from 2.8 to 8.3 m/s, and the predominant wind direction is west^42^. Main characteristics of the sampling sites are shown in Table 2. The study area is characterized by large, open spaces, with gentle slopes. Given the great distance between sites and the complexity at the landscape scale, it is not possible to give a detailed description of the topography at the sampling sites. A general scheme was proposed according to the objectives of this work (Fig. 5b).

To describe the position of the sampling sites within the landscape, two criteria were used: one to describe the location of the sampling site from a topographic point of view (concave, convex or plane), and another one to describe the position of the site from a wind-exposure standpoint, considering the prevailing wind direction (windward, leeward or open).

From an aeolian-transport point of view, convex places are considered as deflation areas, and concave places as accumulation areas. However, the constant winds and the grazing pressure in this region make difficult the identification of net accumulation areas.

### Sampling plots

Fig. 5a shows a scheme of a vegetation sampling plot, and a picture of the studied area. The typical arrangement of the vegetation in patches with interspaces of uncovered soil can be seen in the picture. The picture was taken before the ash fall.

A cluster of 3 BSNE samplers^32^ was placed downwind from every plot. The samplers were placed on a rotating mast, at different heights above the ground (0.15 m, 0.5 m and 1.5 m). The absolute efficiency of the BSNE ranges between 85% and 95%^32^,^33^. Similar types of BSNE arrangements were used for measuring wind erosion in rangelands^43^. A simplified scheme was used for aeolian transport of volcanic material^3^ and also for fresh volcanic ash^16^. A picture of a BSNE mast at a plane, open site, is shown in Fig. 5.

The typical erosion-deposition pattern created by the presence of plants can be seen in the photograph. The effect of vegetation on the aeolian transport of sediment is generally assessed by considering the type and amount of vegetation cover^44^. More theoretical approaches use the drag partition scheme^35^,^36^. The essential parameters of these two frameworks were calculated for the each sampling site (Table 1). Vegetation measurements were made windward from each BSNE cluster, along three transects of 50 m each. This field layout is based on the Australian WARMS method^45^. A very detailed description of the methods used herein, is provided by Oliva *et al*.^46^.

### Particle size distribution of the ash

The ash collected at sampling site 3 (S3) during a period of high mass transport rates (03 august to 23 september 2011) was analyzed with a Malvern Mastersizer 2000. Mean grain diameter at 13.5 cm height was 61 μm, 80% of the material ranged between 11 and 171 μm, showing a poorly sorted (leptokurtic) distribution. Slight differences were found in the particle size distributions across the sampling height. Concerning the distribution over the height, similar findings were reported^16^ for an extreme wind erosion event. However, findings reported in Iceland refer to a much coarser material collected up to 120 cm above the ground level (76% of the material between 0.25 and 1 mm). Size distribution and physical properties across the height are very interesting subjects. However they need further study and comprehensive analysis, and this is not within the aims of this study.

### Mass flux and mass transport calculation

The ash trapped inside the samplers was periodically collected and weighted at the laboratory. Mass values collected at each height were divided by the area of the entrance of each BSNE for obtaining the mass flux *q* (kg m^−2^):


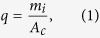


where *m*_*i*_ is the mass collected and *A*_*c*_ is the area of the opening of the trap. The obtained mass fluxes at different heights were fitted to a linear spline interpolation model of the form:


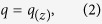


where q is the mass flux at height z.

Finally, the horizontal mass transport per unit width Q (kg m^−1^) was obtained numerically integrating Eq. (2) from zero to 1.5 m height:


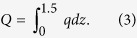


Integration of the mass fluxes over the height has been a widely used method^47^,^48^. Exponential functions are generally used to describe the vertical profile of the mass flux, but vertical profiles in vegetated environments can be much more complicated^25^. Linear spline interpolation is a method used in many research fields, and it could be considered as a feasible method when analyzing field data, especially complex vertical profiles, where ordinary regression may fail. Despite being robust, this calculation method coupled with a three-height sampling arrangement produced a 45% underestimation of the mass transport on unvegetated fields^34^. The software used for the interpolation and integration procedure was Curve Expert ^®^. The resulting value from Eq. (3) was divided by the number of days of the measurement period, obtaining the average daily horizontal mass transport Q, in kg m^−1^ day^−1^.

### Description of the vertical mass-flux profile

The vertical distribution of the mass flux of sediment in vegetated areas is a less studied issue. The vertical profiles of the horizontal mass flux over smooth surfaces, are generally described fitting the measured data to non-linear functions with one or more parameters^34^,^47^,^48^. As vertical profiles of volcanic ash measured herein were very variable, it was not possible to fit a specific, non-linear mathematical function to every dataset. Hence, the slope of a simple linear regression of the mass flux values across the height was preferred as an indicator of the distribution of the transported mass across a vertical plane perpendicular to the soil, and to the wind:


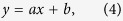


where *y* is the mass flux in kg m^−2^, *x* is the height over the ground in metres and *a* is the slope and *b* is the intercept. This simple method, allowed us to compare the general trend of the profiles obtained at different sites and dates.

### Weather data collection

Meteorological variables were measured at the eastern point of the studied area (Fig. 4). Unfortunately, meteorological data were lost in one case (Table 3). Weather stations in this region are scarce. Long distances, bad road conditions and harsh weather make difficult the functioning and servicing of electronic devices during long measurement periods.

Although weather data is available only for the eastern area of the studied transect, wind is the driving force in aeolian transport, and wind characteristics do not change significantly at a regional scale. On the other hand, the spatial variability of rainfall is very high, and this may have affected the transport values measured at different sites and moments. However, rain events are generally considered to have a short term effect on aeolian transport. Considering the large spatial scale, the long measurement periods, and the descriptive nature of this work, the short term effect of changes in meteorological conditions was neglected.

## Additional Information

**How to cite this article:** Panebianco, J. E. *et al*. Dynamics of volcanic ash remobilisation by wind through the Patagonian steppe after the eruption of Cordón Caulle, 2011. *Sci. Rep.* **7**, 45529; doi: 10.1038/srep45529 (2017).

**Publisher's note:** Springer Nature remains neutral with regard to jurisdictional claims in published maps and institutional affiliations.

## Figures and Tables

**Table 1:** Soil cover, ash depth, flow regime and drag partition parameters.

Site	1	2	3	4	5	6	7
Grass cover (%)	53.8	11.2	11	3.2	13.8	10.2	21
Shrub cover (%)	0	5	15.8	8.6	18.8	18	41.6
Total veg. Cover	67.4	42.2	33.8	17	43.4	28.2	62.6
Estimated ash depth (cm)	4	2.25	4	2.25	2.25	2.25	2.25
Ash depth at nov. 2011 (interpatch area, in cm)	3.38	0.25	0.48	0.45	1.75	0.23	0.38
Mean monthly fractional decrease of the mass transport (σ, %)	8.9	9.4	13.2	5.8	7.5	9.5	12.9
Flow regime (Spacing/height)*	WIF	IRF	IRF	IRF	IRF	IRF	SF
CRE**1**	0.21	0.019	0.059	0.001	0.029	0.01	0.436
S/H**2**	2.6	9.31	4.26	21.1	5.33	8.11	1.8
LC**3**	0.09	0.02	0.05	0.01	0.05	0.02	0.13
BA/FA**4**	0.61	0.74	1.44	2.28	1.71	1.16	2.21

σ: Parameter used for describing supply limited saltation, indicates the exhaustion rate of the particles that can be eroded^20^; IRF: Isolated roughness flow, WF: Wake interference flow, SF: Skimming flow^49^: Concentration of roughness elements = h × b/s^2^; 2: h/s; 3: Lateral cover = ((l × h)/(l + s)^2^); 4: Basal to frontal area ratio = (l × w)/(w × h), where b = basal cover l = patch length, h = patch height, w = patch width, s = interpatch distance.

**Table 2:** Main characteristics of the studied sites.

Site	1	2	3	4	5	6	7
Landscape type	Mountains and hills	Piedmont floodplanes	Mountains and hills	Mountains and hills	Mountains and hills	Basalt plateaus	Basalt plateaus
Relief - Wind exposure	Concave leeward	Plane open	Plane open	Convex open	Concave open	Convex windward	Concave leeward
Longitude	−71.06	−70.97	−70.08	−69.70	−69.71	−69.44	−69.44
Latitude	−41.00	−41.15	−41.25	−41.11	−41.11	−41.76	−41.76
Altitude (masl)	1150	1125	1190	1119	1201	1229	1191
Dominant Species (%cover)	*Festuca pallescens*,	*Festuca pallescens, Acaena splendens*	*Nassauvia glomerulosa, Stipa speciosa*	*Mulinum spinosum, Stipa speciosa*	*Nassauvia glomerulosa, Stipa humilis*	*Nassauvia glomerulosa, Nardophyllum obtusifolium*	*Mulinum spinosum, Poa lanuginosa*

**Table 3:** Weather data recorded at the eastern sites of the transect during the measurement periods. Results are based on hourly data.

Measurement period	27/4/2011	27/6/2011	2/8/2011	22/9/2011	2/11/2011	5/12/2011	2/7/2012	9/10/2012
	27/06/2011	02/08/2011	22/09/2011	02/11/2011	5/12/2011	07/02/2012	9/10/2012	22/11/2012
Wind run	11323.5	8280.9	8449.3	15497.1	12106.0	10736.2	18434.12	nd*
Average hourly wind speed (km h^−1^)	7.5	10.5	12.6	12.6	12.3	13.5	12.0	nd*
Total rain (mm)	26.0	8.6	2.2	34.2	14.4	0.4	43.6	nd*
Relative humidity (%)	64.1	63.9	78.9	73.1	43.2	31.6	29.8	nd*
Direction of highest frequency of maximum wind speeds	NW	NW	NW	WSW	WSW	WSW	NW	nd*
Average maximum hourly wind speed	22.8	20.8	24.1	24.9	24.5	27.5	25.95	nd*
